# Chitinase Chi 2 Positively Regulates Cucumber Resistance against *Fusarium oxysporum* f. sp. cucumerinum

**DOI:** 10.3390/genes13010062

**Published:** 2021-12-27

**Authors:** Jun Xu, Ningyuan Zhang, Ke Wang, Qianqian Xian, Jingping Dong, Xiaohua Qi, Xuehao Chen

**Affiliations:** 1 School of Horticulture and Plant Protection, Yangzhou University, Yangzhou 225009, China; 006963@yzu.edu.cn (J.X.); ningyuan1021@163.com (N.Z.); WK19825301461@163.com (K.W.); QQxian1996@163.com (Q.X.); jpdong@yzu.edu.cn (J.D.); xhqi@yzu.edu.cn (X.Q.); 2 State Key Laboratory of Vegetable Germplasm Innovation, Tianjin 300192, China

**Keywords:** cucumber, transcriptome, chitinases, *Fusarium* wilt, resistance

## Abstract

Cucumber (*Cucumis sativus* L.) is an important vegetable crop worldwide, and *Fusarium* wilt (*FW*), caused by *Fusarium oxysporum* f. sp. cucumerinum (Foc), severely restricts cucumber growth and yield. Accumulating lines of evidence indicate that chitinases play important roles in attacking the invading fungal pathogens through catalyzing their cell wall degradation. Here, we identified the chitinase (Chi) genes in cucumber and further screened the *FW*-responsive genes via a comparative transcriptome analysis and found that six common genes were predominantly expressed in roots but also significantly upregulated after Foc infection. Expression verification further conformed that *Chi2* and *Chi14* were obviously induced by Foc as well as by hormone treatments, compared with the controls. The purified Chi2 and Chi14 proteins significantly affected the growth of Foc in vitro, compared with the controls. Knockdown of *Chi2* in cucumber by virus-induced gene silencing (VIGS) increased susceptibility to *FW*, compared with the *Chi14*-silenced and control plants, and silencing of *Chi2* drastically impaired gene activation in the jasmonic acid pathway, suggesting that the *Chi2* gene might play positive roles in cucumber *FW* defense and, therefore, can provide a gene resource for developing cucumber-*FW*-resistance breeding programs.

## 1. Introduction

Cucumber (*Cucumis sativus* L.) is an economically vegetable crop and cultivated worldwide [1]. However, the growth and production of cucumber are seriously constrained by cucumber *Fusarium* wilt (*FW*), which is a typical soil-borne and destructive fungal disease caused by *Fusarium oxysporum* f. sp. cucumerinum (Foc) [2,3,4,5,6].

Cucumber *FW* is usually considered one of the most significant biotic factors limiting global cucumber output and quality, resulting in the wilting of leaves, or even the entire plant, and ultimately plant death within several days or weeks after pathogen infection [7]. Fearfully, this disease can hazard the entire growth and development stages of cucumber [3]. Troublingly, *FW* is extremely difficult to control because Foc can survive in the soil, straw, or seeds for many years or even decades, executing the disease cycles for a primarily long term [8]; additionally, changes in pathogenicity have led to the ineffectiveness of certain fungicides [9,10]. Using resistant cultivars to mine more disease resistance genes and investigate disease resistance mechanisms is recognized as the most sustainable way to control *FW* disease.

Chitinases function in the hydrolysis of the β-1-4 linkage in the N-acetyl-D-glucosamine polymer of chitin, catalyzing fungal cell walls degradation, and are found and applied for defense by directly attacking invaded fungal pathogens in various plants [11,12]. Glycoside hydrolase (GH) families 18 and 19 are defined as chitinases based on the sequence similarity of the conserved catalytic domains [13]. Additionally, plant chitinases are recognized as pathogenesis-related (PR) proteins, determined as PR-3, PR-4, PR-8, or PR-11, respectively [14], which participate in plant immunity. Practically, plant chitinases are more propitious to inhibit the reproduction of fungi by generating hypersensitive reactions and defense responses [15]. Over-expression of chitinase genes from several plant species has successfully enhanced resistance to fungal pathogens in different transgenic plants [16,17,18,19]. However, to date, the functions of chitinase genes in cucumber have not been systematically analyzed, and the resistance of these transgenic lines against pathogens remains unclear.

An increasing number of studies focus on functional genomics to analyze the host immunity to restrict disease invasion [20]. Hence, the availability of data on the whole genome of cucumber species [1,21,22] made it possible to systematically investigate the functions of chitinase genes and identify the *FW*-resistance genes in cucumber. In this study, we performed a comparative transcriptome analysis and determined that six chitinase genes were predominantly expressed in cucumber roots and also significantly upregulated after Foc infection. Expression verification further found that *Chi2* and *Chi14* were obviously induced by Foc, as well as the hormone treatments, compared with the controls. The results of an anti-fungal assay in vitro and gene-silencing method in the cucumber confirmed that *Chi2* genes play positive roles in cucumber *FW* defense. These findings might provide effective gene resources for improving cucumber *FW* resistance through breeding programs.

## 2. Materials and Methods

### 2.1. Plant Materials and Treatments

Cucumber inbred line “Rijiecheng” exhibited resistance to Foc used in this study. Then, the cucumber seedlings were grown in a growth chamber under 16 h/8 h with 25 °C/18 °C during day/night, respectively.

Foc was propagated on potato dextrose agar (PDA) medium at 28 °C for 4 days and then cultured in potato dextrose broth (PDB) medium on a shaker at 180 rpm at 28 °C for 3 days. Next, the spore suspension was adjusted to 10^6^ spores/mL using sterile-distilled water for inoculation of the cucumber seedlings via the dip inoculation method [6]. Seedlings treated with sterile water were used as mock inoculation controls. The roots of cucumber seedling were harvested at different time points (0, 24, 48, 96, 192 h) after the Foc and mock treatment with three repeats. Moreover, the seedlings were, respectively, induced with 1 g/L ethephon, 100 µM/L methyl jasmonate (MeJA), and 100 µM/L salicylic acid (SA), at time points containing 0, 6, 12, and 24 h. Additionally, all samples were rapidly frozen in liquid nitrogen and stored at −80 °C until analysis.

### 2.2. Identification of the Chitinase Genes in Sequenced Cucumber 

The available genomic database of sequenced cucumber was downloaded from http://cucurbitgenomics.org/, accessed on 17 December 2021. The Pfam protein family databases including the Glyco_hydro_18 (PF00704) and Glyco_hydro_19 (PF00182) domains [23], and the HMMER software version 3.0 were used to identify the chitinase genes in the cucumber species [24]. Further, SeqHunter software version 1.0 was used to isolate the chitinase genes from the cucumber databases. All the selective genes were further confirmed by the databases including the Pfam protein family, SMART, and National Center for Biotechnology Information (NCBI). The distribution of the identified chitinase genes in cucumber was visualized by MapInspect software, where the chromosome (Chr) orders from *Chr1* to *Chr7* referred to the map [22]. The chitinase genes in cucumber were named *Chi 1* to *Chi 27* based on the order of their chromosomes and location.

### 2.3. Transcriptome Analysis and Candidate Gene Identification 

Our previous studies constructed the transcriptome of cucumber roots inoculated with Foc at 0, 24, 48, 96, and 192 h (NCBI Accession No. PRJNA472169). The differentially expressed genes (DEGs) were identified using the DESeq R package [25]. Additionally, the transcriptome of the cucumber with various tissues and organs was downloaded from the database (http://cucurbitgenomics.org/rnaseq/cu/3, accessed on 17 December 2021). The two transcriptomes were used for the identification and comparative analysis of chitinase genes, respectively.

### 2.4. Quantitative Reverse-Transcription PCR (qRT-PCR) Analysis

Total RNA of the cucumber roots inoculated with Foc at different time points (0, 24, 48, 96, and 192 h), and the samples with the hormone treatments, which were isolated using a TaKaRa MiniBEST Plant RNA Extraction Kit (TaKaRa, Dalian, China). Then, RNA was treated with DNase I to remove genomic DNA, and next, 2 μg RNA was used for the synthesis of the first-strand cDNA via the Superscript first-strand synthesis system (Invitrogen Life Technologies, Carlsbad, CA, USA). The AceQ SYBR Green Master (Vazyme, Nanjing, China) was used in accordance with the manufacturer’s instructions, with three technical replicates for each biological sample. The qRT-PCR was performed using an Iqtm5 Multicolor qPCR detection system (BioRad, Rockville, MD, USA). The Beacon Designer 7.0 software was used to design primer pairs according to the chitinase gene sequences in cucumber. The cucumber tubulin alpha chain gene (*Csa4G000580*) acted as the reference gene; all PCR primer data are available in Supplementary Materials, Table S1.

### 2.5. Chi2 and Chi14 Protein Purification 

*Chi2* and *Chi14* were cloned from cDNA of cucumber roots and then constructed the *pet28a* vectors for sequencing verification. Subsequently, the *Chi2*-*pet28a* and *Chi14*-*pet28a* vectors were introduced into the Rosetta competent cells (DE3), and positive clones were cultured in 5 mL liquid LB medium containing 50 μg/mL kanamycin antibiotic on a shaking table with 160 rpm per minute until growing to an OD_600_ of 0.6 at 37 °C. A portion of bacterial cells was taken out as a control group, and the remaining bacteria solution was added to the IPTG inducer (final concentration 1 mM) shocking for 4 h at 37 °C. Then, 0.15 mL bacterial liquids were taken from two groups, and bacterial cells were re-suspended with 40 μL 1 × loading buffer after centrifugation and further detected with SDS–PAGE. 

Next, 100 mL above bacterial cells was added to a 2000 mL LB medium containing 50 μg/mL kanamycin antibiotic, with 160 rpm per minute until growing to an OD_600_ of 0.6 at 37 °C. Subsequently, the IPTG inducers were added to the bacterial cells, with a final concentration of 0.1 mM, and shaken continuously for 8 h at 30 °C. After centrifugation at 8000× *g* for 3 min, the collected bacterial cells were resuspended in 50 mL pre-cooling NTA-0 buffer, and lysozyme was added on ice, with a final concentration of 0.1 mg /mL, for 30 min. The supernatant and precipitate were separated and collected after obtaining the sonicated cells by the ultrasonic crusher, and 10 μL supernatant and precipitate were taken for SDS–PAGE detection; the remaining supernatant and precipitate were stored at 4 °C until analysis. Finally, the above precipitates were purified and further tested by Western blot as described by Yakushiji et al. (2004) [26]. The commercialized protein TFPI (CUSABIO, Wuhan, China), added with His-tag, was 35 kDa and used as a control (CK), to distinguish the molecular weight of the Chi2-His and Chi14-His proteins.

### 2.6. Functional Characterization of Chi2 via Virus-Induced Gene Silencing (VIGS)

The VIGS vector (*pV190*) derived from cucumber green mottle mosaic virus (CGMMV) were used for functional verification of target genes generously provided by Dr. Qinsheng Gu of Zhengzhou Fruit Research Institute, Chinese Academy of Agricultural Sciences (Zhengzhou, China), and these vectors produced very mild viral symptoms and efficiently triggered gene silencing in the cucumber [27]. *pV190*: *CsPDS*, where *CsPDS* (*Csa4G011080*) encoded phytoene desaturase, was used as a control, to assess the silencing efficiency based on the photobleaching phenotypes. 

*pV190* vector was used to construct *pV190: Chi2* and *pV190: Chi14* to silence target genes, which contained 300 base pair (bp) gene-specific fragments of *Chi2*, and 340 bp gene-specific fragments of *Chi14*, respectively. These vectors were introduced into *Agrobacterium* *tumefaciens* strain GV3101 by freeze–thaw transformation, and then positive clones were transferred into LB liquid media containing the antibiotics 50 μg/mL kanamycin and 25 μg/mL rifampicin on a shaking table with 160 rpm per minute to grow to an OD_600_ of 0.5 at 28 °C. Following centrifugation, the *Agrobacterium* cells were resuspended by inducing a solution containing 10 mM/L MgCl_2_, 10 mM/L MES, and 100 µM/L acetosyringone to adjust the final OD_600_ value to 0.8–1 and maintained at room temperature (25 °C) for more than 2 h before inoculation. Subsequently, the *Agrobacterium* cells were infiltrated into the fully expanded cotyledons of cucumber seedlings. All plants were grown in the same growth chamber at 25/18 °C (day/night), with a16 h light/8 h dark cycle, and changes in plant phenotypes were observed. 

After two weeks, the *pV190: CsPDS*-infiltrated plants showed highly uniform bleaching in newly emerged leaves. Meanwhile, we harvested the leaves from at least three seedlings per treatment to verify the silencing effect of these vectors via comparing expression levels of candidate genes in controls and gene-silencing plants. All VIGS cucumber seedlings were dip inoculated with Foc conidia suspension (1 × 10^6^ spores/mL), as described previously [6], to analyze disease phenotype for the identification of gene functions. Each treatment was applied to more than 20 plants, and the VIGS experiments were repeated at least three times to increase the liability of the results.

### 2.7. Histochemical Staining and Microscopic Observations

We also used the VIGS leaves to detect the pathogenesis after Foc infection. The histochemical detection of cells death in the Foc-infected leaves was also carried out through trypan blue staining, as described by Yoshioka et al. (2003) [28]. Infected leaves were transferred to a trypan blue solution containing 10 mL of lactic acid, 10 mL of glycerol, 10 g of phenol, 10 mL of H_2_O, and 10 mg of trypan blue, and further diluted in ethanol 1:1 and were boiled for 1 to 2 min. The Foc-infected leaves were then destained overnight in chloral hydrate to remove the green background followed by photographing with a stereoscopic microscope (SteREO Discovery.V8, ZEISS, Oberko, Germany). 

### 2.8. Statistical Analysis 

The statistics were analyzed using the SPSS Statistical 20.0 software, and the differences between different groups were calculated with Student’s *t*-tests.

## 3. Results

### 3.1. Expression Profiles of Chitinase Genes in Different Cucumber Tissues

**Figure 1:**
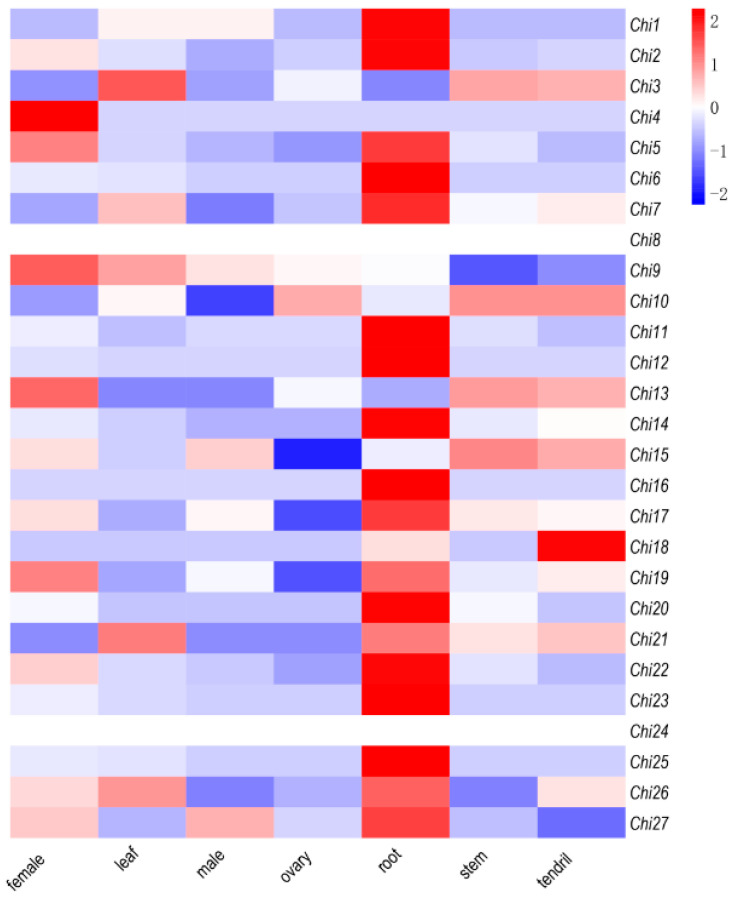
Transcriptional analysis of chitinase genes in cucumber tissues and organs. Female (female flower tissue), leaf, male (male flower tissue), ovary, root, stem, and tendril were used for the expression analysis. The expression values were converted with Log_2_ (FPKM) to calculate the expression levels of *Chi*s. The differences are displayed in colors indicated with a scale. This RNA-Seq data can be downloaded with NCBI Accession Number PRJNA80169.

We identified 27 chitinase genes (*Chi*s) with verified domains, and these genes were matched to 6 scaffolds in cucumber, except in chromosome 7, and preferentially named from *Chi1* to *Chi27*, based on the reordered cucumber chromosomes orders and location (Table S2). To further investigate the roles of chitinase genes in cucumber, transcriptome data from cucumber tissues containing the female flower tissue (female), leaf, male flower tissue (male), ovary, root, stem, and tendril were used to analyze the gene expression patterns [1]. In total, 27 *Chi*s were found to be expressed in the different tissues and organs, indicating that they might play important roles in the diverse developmental and spatial regulation in cucumber (Figure 1). Among them, *Chi4*, *Chi9,* and *Chi13* were predominantly expressed in female organs, and additionally, *Chi10* and *Chi15* were constitutively expressed at high levels in various tissues and organs. Moreover, 10 *Chi*s were predominantly expressed in root tissue of cucumber, which is the first tissue that soil-borne pathogens contact and invade [29,30]. These results showed that these cucumber *Chi*s possessed overlapping but diverse expression characteristics in various tissues and organs, indicating that they might have conserved structures but different functions.

### 3.2. Transcriptome Analysis and Expression Verification of Chitinase Genes in Response to Foc 

**Figure 2:**
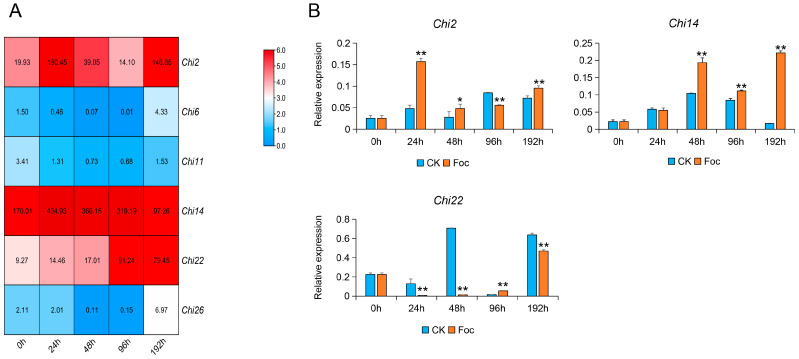
Transcriptional analysis and verification of chitinase genes: (**A**) transcriptional analysis of the chitinase genes and visualized by hierarchical clustering. Fold difference was designated as Log_2_ (FPKM) to calculate *Chi*s expression levels with the colors indicated in the scale. Sampling time points include 0 h, 24 h, 48 h, 96 h, and 192 h after Foc inoculation; (**B**) verification of candidate gene expression in response to Foc in cucumber. Induction of candidate DEGs after inoculation with Foc as assessed by qRT-PCR. The error bars with standard deviations were calculated from three biological replicates. * indicates significantly different at *p* ≤ 0.05; ** indicates significantly different at *p* ≤ 0.01.

To obtain further evidence of the role of these chitinase genes in response to Foc, we used the transcriptome of cucumber roots inoculated with Foc at 0, 24, 48, 96, and 192 h to analyze the expression patterns of the chitinase genes. As a result, six chitinase genes were significantly induced by Foc, and among them, *Chi2*, *Chi14*, and *Chi22* had higher expression levels (Figure 2A). To confirm the relationship between chitinase genes and Foc tolerance in cucumber, the candidate chitinase genes were verified in Rijiecheng cucumbers inoculated with Foc. It was observed that *Chi2*, *Chi14*, and *Chi22* had significantly induced expression by Foc (Figure 2B), and *Chi2* and *Chi14* showed upregulated expression tendency, compared with the controls, indicating that they might play important roles in cucumber defense responses against *FW*.

### 3.3. Expression Analysis of Chi2 and Chi14 in Response to Hormone Signals

**Figure 3:**
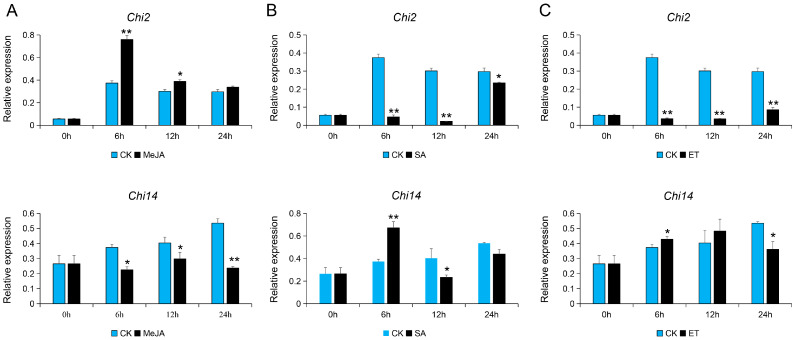
The expression patterns of *Chi2* and *Chi14* in cucumber treated with hormones: (**A**–**C**) the cucumber roots were inoculated with hormones including MeJA, SA, and ET, respectively, and were used to assess the expression of *Chi2* and *Chi14* by qRT-PCR. The error bars with standard deviations were calculated from three biological replicates. * indicates significantly different at *p* ≤ 0.05; ** indicates significantly different at *p* ≤ 0.01.

Accumulated lines of evidence show that plant hormones played important roles in regulating developmental processes and signaling networks involved in plant responses to a wide range of biotic stresses [31]. The expression patterns of chitinase genes were also regulated by a variety of plant phytohormones including JA, SA, ET, etc., which contributed to the disease resistance in plants [32]. Additionally, based on the transcriptional analysis of cucumber roots inoculated with Foc, we found that many DEGs enriched in the plant hormone signal transduction mainly related to the JA, ET, and SA [6]. Hence, we analyzed the expression patterns of *Chi2* and *Chi14* in cucumber samples treated with SA, MeJA, and ethephon. As a result, the transcription levels of *Chi2* and *Chi14* were significantly induced by these three hormone treatments. Among them, *Chi2* was also significantly upregulated and reached its peak 6 h after MeJA treatment but was downregulated after using SA and ET treatments. Likely, *Chi14* was also affected by MeJA, SA, and ET; specifically, the expression levels of Chi14 was increased and accumulated at 6 h after SA and ET treatments, compared with the control; however, it was also significantly downregulated at 6–24 h after MeJA, as well as the ET treatment at 24 h (Figure 3). These results imply that *Chi2* and *Chi14* may participate in JA, SA, and ET signaling pathways to protect plants from various stresses.

### 3.4. Chi2 and Chi14 Suppresses Growth of Foc In Vitro

**Figure 4:**
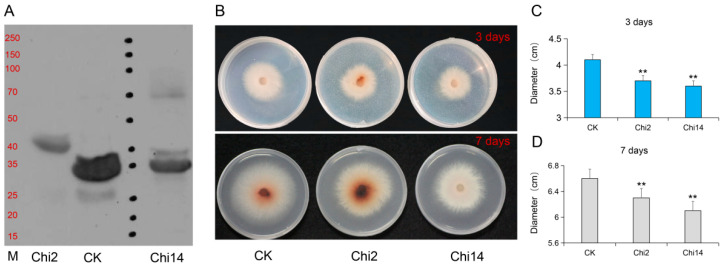
Chi2 and Chi14 affect the growth of Foc in vitro: (**A**) detection of these two purified proteins by Western blot with anti-His. Lane Chi2, recombinant His-Chi2; lane CK, the 35 kDa protein TFPI (CUSABIO, Wuhan, China) added with the His-tag; lane M, marker, molecular quantity standard (250/150/100/70/50/40/35/25/20/15 kDa); lane Chi14, recombinant His-Chi14; (**B**) the phenotypic differences between the Foc strain cultured on PDA medium supplemented with sterile water (CK), Chi2, and Chi14 protein solutions at 3 and 7 days after inoculation; (**C**,**D**) the diameter of Foc grown on CK, Chi2, and Chi14 protein solutions at 3 and 7 days after inoculation. The error bars with standard deviations were calculated from three biological replicates. ** indicates significantly different at *p* ≤ 0.01.

The entire open reading frame of Chi2 and Chi14 were cloned into a *pet28a* vector fused with a His-tag and further expressed in *Escherichia coli*. We used the 35 kDa protein TFPI (CUSABIO, Wuhan, China) added with the His-tag as the control. Subsequently, we detected the purified recombinant proteins by Western blot with anti-His and found that Chi2 and Chi14 were 44 KD and 37 KD, respectively (Figure 4A). To ascertain the functions of the Chi2 and Chi14 in cucumber *FW* defense, we further cultured the Foc strain on the PDA medium supplemented with sterile water (CK) and Chi2 and Chi14 recombinant protein solutions to observe phenotypic differences at the 3 and 7 days after inoculation (Figure 4B). The Foc growth on PDA supplemented with Chi2 and Chi14 protein solutions was obviously suppressed, compared with control samples (Figure 4C), indicating that Chi2 and Chi14 might play important roles in defense against Foc infection in cucumber.

### 3.5. Silencing Chi2 Impairs Cucumber Tolerance to Foc

**Figure 5:**
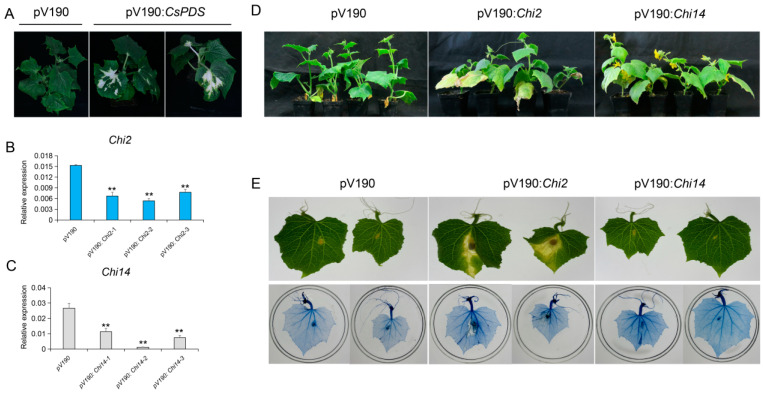
Silencing of *Chi2* significantly impaired the resistance to Foc in cucumber: (**A**) silencing of the endogenous *PDS* gene in cucumber through VIGS. The leaf bleaching phenotype was observed three weeks after infiltration in *pV190: CsPDS* plants; (**B**,**C**) the transcription levels of *Chi2* and *Chi14* in the *Chi2*-silenced, *Chi14*-silenced, and *pV190* leaves; the error bars with standard deviations were calculated from three biological replicates; ** indicates significantly different at *p* ≤ 0.01; (**D**) cucumber phenotypes at 14 days after Foc inoculation; the VIGS plants were dip inoculated with Foc conidia suspension (1 × 10^6^ conidia/mL); (**E**) the phenotypes of the *Chi2*-silenced, *Chi14*-silenced, and *pV190* leaves inoculated with Foc and trypan blue staining. The leaves were infected with Foc for 5 days and performed the trypan blue staining and microscopic observation.

To further investigate the functions of *Chi2* and *Chi14* in Foc resistance, we constructed *pV190*: *Chi2*, *pV190*: *Chi14*, and *pV190*: *CsPDS* vectors to silence endogenous genes in cucumber. All constructs were infiltrated into cotyledon, and two weeks later, all cucumber seedlings infiltrated with *PV190*: *CsPDS* showed bleached leaf phenotype in newly emerged leaves, suggesting that the *PDS* gene was silenced in these seedlings (Figure 5A). Subsequently, the VIGS seedlings infiltrated with *pV190*: *Chi2*, *pV190*: *Chi14*, and *pV190* constructs were used for verification of gene-silencing effects via the qRT-PCR method. We found that the transcripts of *Chi2* and *Chi14* were significantly downregulated, compared with *pV190* treatments (*p* < 0.01) (Figure 5B,C). 

After *Chi2* and *Chi14* were silenced, we inoculated cucumber seedlings with Foc by dip infection, with a final concentration of 1 × 10^6^ spores per milliliter. Three weeks after inoculation, the *Chi2*-silenced seedlings showed discoloration of stem bases and possessed obviously leave wilting phenotype, compared with the cucumber samples infiltrated with *pV190*: *Chi14* or *pV190* vectors (Figure 5D). We also used the leaves of VIGS seedlings infiltrated with *pV190*: *Chi2*, *pV190*: *Chi14*, and *pV190* constructs, to analyze the pathogenesis and death cells after Foc infection. We further found that the *Chi2*-silenced leaves were more susceptible to Foc infection, and also, the trypan blue staining showed that *Chi2*-silenced leaves had deep color and dead cells in the points inoculated with Foc (Figure 5E). Furthermore, we found that gene expression in the JA pathway included a basic helix-loop–helix-leucine zipper transcription factor (MYC2), jasmonate-associated VQ motif gene1(*JAV1*), and 2-oxophytodienoic acid reductases (OPR) 1 were significantly down-regulated in the *Chi2*-silenced leaves (Figure S1). These findings confirmed that down-regulated expression of *Chi2* impaired cucumber tolerance to Foc, and *Chi2* genes might play important roles in cucumber *FW* defense.

## 4. Discussion

*FW* acts as a destructive vascular disease; however, the related mechanisms of *FW* suppression are largely unknown. Chitinases functions by catalyzing the hydrolysis of chitin and damaging fungal cell walls, which are found and applied for defense against fungus pathogens in various plants. Although the genome-wide investigation of chitinase genes has been reported in many plant species, to date, the identification or functional analysis of chitinase genes involved in *FW* defense in cucumber remains limited. The number or distribution of chitinase genes shows obvious differences in different plants. For instance, 24 chitinase genes have been found and distributed between all 5 of the chromosomes in *Arabidopsis*, while rice chitinase genes are positioned on all chromosomes except chromosome 7 [33]. In cucumber, chitinase genes have been identified, and their evolutionary characteristics and expression patterns have also been analyzed in response to *F. oxysporum* [34]. Data on recent advances regarding the whole genome of cucumber species [1,21,22] made it possible to conveniently investigate the functions of chitinase genes. Here, we systematically identified 27 candidate chitinase genes in the sequenced cucumber species and further found that these genes were distributed on all chromosomes except chromosome 7 (Table S2).

An increasing number of studies found that the expression characteristics of chitinase genes exhibited both constitutive and induced patterns [35,36,37,38]. Among them, induced expression levels of chitinases have been recorded in response to numerous abiotic stresses containing wounding, drought, salinity, cold, heavy metals, ozone, frost, and UV light [37,38], as well as biotic stresses, including infectious fungi [17,39]. The availability of the whole genome of cucumber species made it convenient to screen candidate genes participating in plant growth and development, as well as abiotic and biotic stresses. For example, to investigate the development of mesocarp in cucumber, the expression profiles of mesocarp at 0, 3, 6, and 9 days after pollination (DAP) by RNA sequencing is used to identify functional genes [40]. An integrated analysis of the miRNA-seq and RNA-seq was performed to identify the *FW*-responsive miRNAs and their target genes in the *FW*-resistant cucumber cultivar Rijiecheng [41]. Here, we performed a comparative transcriptome analysis of the cucumber tissues (female, leaf, male, ovary, root, stem, and tendril) and roots of *FW*-resistant inbred line Rijiecheng infected with the Foc to screen the chitinase genes (Figure 1 and Figure 2). The expression verification found that *Chi2* and *Chi14* were significantly upregulated affected by Foc. An increasing number of studies show that plant hormones play important roles in defense against a wide range of biotic stresses [31], and the expression patterns of chitinase genes are also regulated by a variety of plant phytohormones, including JA, SA, ET, etc. [32]. Furthermore, transcriptional analysis of cucumber roots inoculated with Foc shows that many DEGs enriched on the plant hormone signal transduction mainly related to the JA, ET, and SA [6], suggesting that these hormones play important roles in *FW* defense. Here, we found that *Chi2* was quickly upregulated, influenced by the Foc and MeJA in cucumber, therefore affecting disease resistance. Further research needs to define the precise underlying functions or molecular mechanisms of *Chi2* in *FW* resistance through transgenic expression analysis in cucumber cultivars. Here, we found that *Chi 2* and *Chi14* were significantly upregulated, as a result of the effects of MeJA and SA, respectively (Figure 3). The diverse expression patterns of candidate genes might be affected by the concentration gradient of hormone and time points of treatments, which suggested the chitinase genes possessed diverse but overlapping functions.

Chitinases suppress fungal pathogens by affecting the fungal cell wall degradation. Several chitinase genes from different plants or fungi have been identified and successfully applied in different crops for disease defense. For instance, *Cht-2* and *Cht-3* are expressed in the different tissues and organs in the japonica rice, affecting the resistance to blast (*Magnaporthe grisea*) [17]. In pepper plants, co-expression of the *ChitIV* and receptor-like cytoplasmic protein kinase (*CaPIK1*) significantly enhances the *CaPIK1*-triggered cell death response, the abundance of reactive oxygen species (ROS), and resistance to *Xanthomonas campestris* pv. vesicatoria (*Xcv*) infection [42]. Taken together, these studies suggested that chitinase genes might play particular roles in the defense against fungal pathogens. Here, we also analyzed the inhibitory effect of Foc influenced by the Chi2 and Chi14 proteins with three repeats at different time points and found that Chi2 and Chi14 obviously suppress the growth of Foc, compared with the control (Figure 4). The function difference between *Chi2* and *Chi14* were further analyzed through the VIGS technology, and it was found that *Chi2*-silenced leaves were more susceptible to Foc infection (Figure 5). Although *Chi2* and *Chi14* are predominantly expressed in roots and obviously induced by the Foc, the expression of *Chi2* plays a more considerable role in *FW* defense, compared with *Chi14.* The expression levels of *Chi 2* are obviously upregulated after MeJA treatment, but *Chi14* possesses the converse tendency and is upregulated after SA treatment. In other host plants, SA and JA participate in activating the complex defense signaling networks [31,43]. For instance, JA acts with ET to mediate resistance against necrotrophic pathogens, whereas SA affects defensive responses to effectively control hemi-biotrophs and biotrophs, as well as inducing the systemic acquired resistance [44,45]. Accumulated lines of evidence show that many induced genes related to JA, including *CYP82D* [46], *GaWRKY1* [47], *GhJAZ2,* and *GhPDF1.2* [48], positively contribute to the disease resistance of cotton to a soil-borne fungal pathogen *Verticillium* *dahliae*. Likely, we found that the *MYC2*, *JAV1,* and *OPR1* genes in the JA pathway were significantly down-regulated in the *Chi2*-silenced leaves, and it is speculated that *Chi2* may participate in regulating the JA pathway and have a positive role in regulating cucumber resistance against *FW* infection. Furthermore, although the VIGS method could deal with the phenotype of silent plants quickly, the silencing efficiency and lasting time of the phenotype were still limitations affecting the analysis of the functions of candidate genes in the later period of cucumber. In future studies, we will obtain the transgenic lines through over-expressing and knockdown of *Chi2* and *Chi14* in cucumber to better determine the molecular mechanisms or regulatory networks of *FW* defense and develop resistant cultivars.

## 5. Conclusions

Cucumber *Fusarium* wilt (*FW*) severely restricts cucumber yields and profits; however, the resistance genes and mechanisms underlying the *FW* tolerance in cucumber are poorly understood. An increasing number of studies found that chitinases played important roles in attacking the invading fungal pathogens through catalyzing their cell wall degradation. In this study, we performed a comparative transcriptome analysis and expression verification, and the results revealed that *Chi2* and *Chi14* were obviously induced by Foc, as well as hormone treatments, compared with the controls. We also found that Chi2 and Chi14 purification proteins significantly affected the growth of Foc in vitro compared with the control. Silencing of *Chi2* resulted in increased susceptibility to *FW*, compared with the *Chi14*-silenced and control plants, suggesting that the *Chi2* gene might play positive roles in cucumber *FW* defense and, therefore, can provide gene resources to develop cultivars resistant to cucumber *FW*.

## Data Availability

The RNA-Seq data of cucumber roots inoculated with Foc, and cucumber tissues and organs associated with this study were downloaded with NCBI Accession Number PRJNA472169 and PRJNA80169, respectively.

## Supplementary Materials

The following are available online at https://www.mdpi.com/article/10.3390/genes13010062/s1, Table S1: Information of the PCR primers, Table S2: The cucumber chitinase genes, Figure S1: *Chi2* involved in activation of JA-related genes.
