# Supplementary figures and images for: Chitinase Chi 2 Positively Regulates Cucumber Resistance against Fusarium oxysporum f. sp. cucumerinum
Source: Genes (Basel). 2021 Dec 27;13(1):62. doi: 10.3390/genes13010062 (PMC8775131; doi:10.3390/genes13010062)

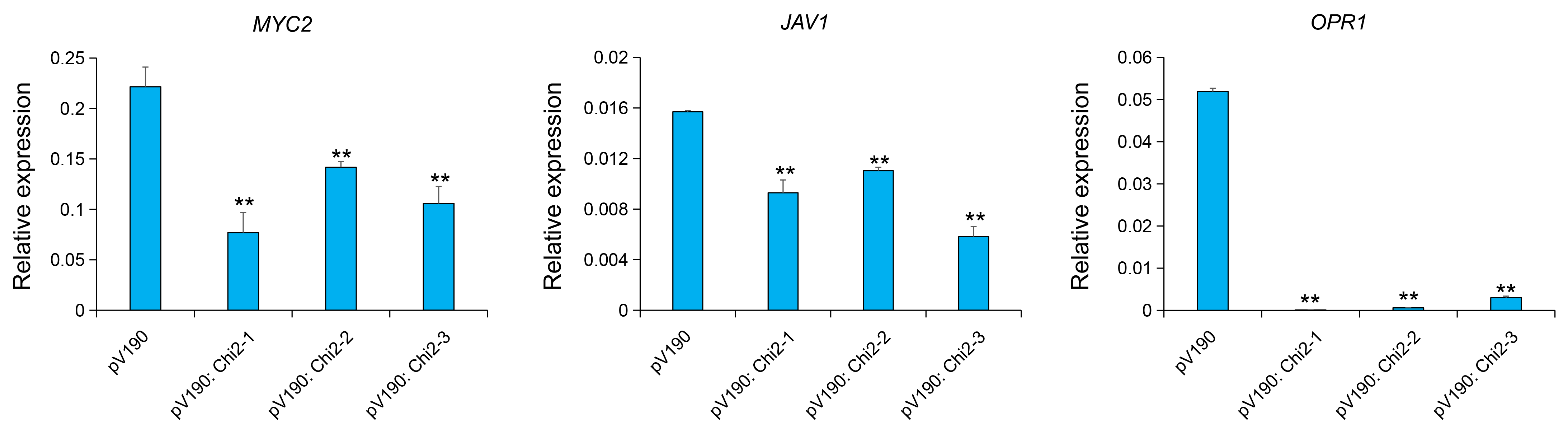

Supplement: Supplementary file 1 [file genes-13-00062-s001.zip › genes-1479786-supp/Figure S1 Chi2 involved in activation of JA-related genes.tif]
